# Inspecting the electronic structure and thermoelectric power factor of novel p-type half-Heuslers

**DOI:** 10.1038/s41598-021-00314-6

**Published:** 2021-10-21

**Authors:** Shakeel Ahmad Khandy

**Affiliations:** Department of Physics, National Taiwan University, Taipei, 10617 Taiwan, ROC

**Keywords:** Thermoelectric devices and materials, Materials science, Physics

## Abstract

In line for semiconducting electronic properties, we systematically scrutinize the likely to be grown half-Heusler compounds *XTaZ *(*X = Pd, Pt and Z* = *Al, Ga, In*) for their stability and thermoelectric properties. The energetically favored *F-43m* configuration of XTaZ alloys at equilibrium lattice constant is a promising non-magnetic semiconductor reflected from its total valence electron count (*N*_*V*_ = 18) and electronic structure calculations. Alongside mechanical stability, the dynamic stability is guaranteed from lattice vibrations and the phonon studies. The energy gaps of these stable Ta-based materials with *Z* = *Ga* are estimated to reach as high as 0.46 eV when *X* = *Pd* and 0.95 eV when *X* = *Pt*; however, this feature is reduced when *Z* = *Al/In* and *X* = *Pd/Pt,* respectively. Lattice thermal conductivity calculations are achieved to predict the smallest room temperature value of *K*_*L*_ = 33.6 W/K (PdTaGa) and 38.0 W/mK (for PtAlGa) among the proposed group of Heusler structures. In the end, we investigated the plausible thermoelectric performance of *XTaZ* alloys, which announces a comparable difference for the *n*-type *and p*-type doping regions. Among the six alloys, PtTaAl, PtTaGa and PtTaIn are predicted to be the most efficient materials where the power factor (PF) elevates up to ~ 90.5, 106.7, 106.5 mW*/*(K^2^m), respectively at 900 K; however the lower values are recorded for PdTaAl (~ 66.5), PdTaGa (~ 76.5) and PdTaIn (~ 73.4) alloys. While this reading unlocks avenues for additional assessment of this new class of Half Heuslers, the project approach used here is largely appropriate for possible collection of understandings to realize novel stable materials with potential high temperature applications.

## Introduction

Fast-track discovery of new Heusler semiconductor phases from high-throughput computations and advancement in experimental procedures have endured this giant family to trigger new and active research tactics for potential applications^1–4^. The recent breakthroughs of having superconducting effects^5^, high Curie temperatures^6^, topological effects like Weyl or Dirac phenomena^7,8^, spin varying electronic structure^9,10^, skyrmions and giant anomalous Hall effects^11,12^ intensified the new paradigm of research on such materials. With the advent of Heusler alloys, practical applications in the device fabrication of spin injectors or magnetic tunnel junctions have gained momentum because of the efficiency and durability^13,14^. Heusler based thermoelectric materials in general have become the spotlight with the recent discovery of a thin layered Heusler material Fe_2_V_0.8_W_0.2_Al exhibiting a figure of merit (ZT) equal to 5 or 6^15^. The same dimensionless parameter ZT = S^2^σT/κ, governs the efficiency of a thermoelectric module, where Seebeck coefficient (S), electrical conductivity (σ) and thermal conductivity (κ). However, κ = κ_e_ + κ_l_ is inturn the sum of electronic (k_e_) and phonon (k_l_) contribution parts of thermal conductivity^16^. Recently, the artificial layers of Bi_2_Te_3_ and Sb_2_Te_3_ displayed the largest values of ZT = 2.4 at room temperature^17^; in SnSe, ZT reached a maximum of 2.6^18^, and in p-type PbTe-SrTe, ZT = 2.5^19^. Among Heuslers the TaFeSb^20^ and Ti/Sn doped NbFeSb^21^ have attained ZT equal to 0.5 and 1.1 experimentally. *Kaur *et al.reported theoretically the p-type semiconductor TiPdSn with 0.74 at 500 K^22^ and TaIrSn material with ZT of 0.61 at 900 K^23^, which are only few to report amid the vast database of thermoelectric Heuslers. However, efforts are being made to find the more efficient Heusler thermoelectrics to have increased competence in converting waste heat to direct electricity.

**Figure 1:**
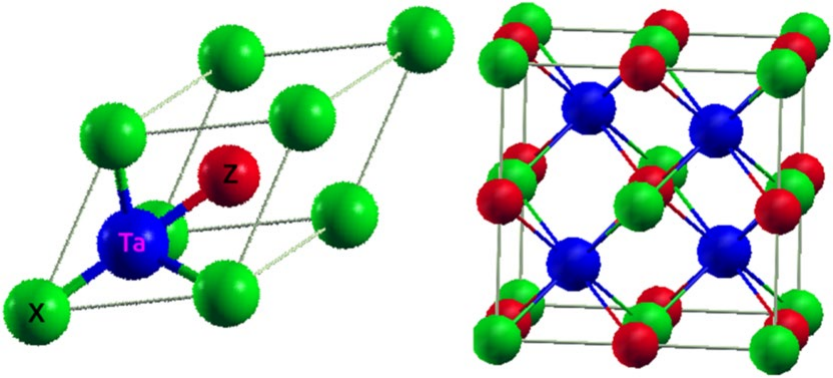
Primitive cell of XTaZ molecule and lattice structure in F-43m configuration. Green balls represent X(Pd/Pt), Blue represent Ta atoms and red spheres represent Z(Al/Ga/In) atoms, respectively.

Half-Heuslers (HH) possess XYZ-type composition, specifically holding non-centrosymmetric C1b cubic structure^24^ within F-43m space group. HH’s can be thought as a full-Heusler (FH) X_2_YZ structure in which D (0.75, 0.75, 0.75) site is empty and the other three sites, A (0, 0, 0), B (0.25, 0.25, 0.25) and C (0.5, 0.5, 0.5) are occupied by Z, Y and X atoms, respectively in a unit cell. The potential feature of electronic structure of such compounds range from half-metallicity^25^ to semiconductor or semimetallic to topological behavior^26,27^. In this article, we examine six half-Heusler compounds with chemical formula XTaZ, where X is (Pd or Pt) atom and Z is a group IIIA (Al, Ga or In) element. The primitive cell of MgAgAs type XTaZ HH’s alongside the occupation sites of individual atoms is depicted in Fig. 1. The total sum of valence electrons in all the six materials is 18, which significances the exhibition of nonmagnetic and semiconducting nature of all these materials. The semiconductor nonmagnets of XYZ kind have been intensively studied, but no theoretical or experimental work has been reported about the present set of materials till date. Since, the electronic composition of this class is mainly 4d/5d electrons, therefore the electrical conductivity is expected to be larger in comparison to 3d HH systems. This also adds to the feature of heavier mases of constituent elements, which in turn is expected to decrease the lattice thermal conductivity. The main objective is to explore the equilibrium electronic structure of these transition-metal atom based Heuslers for the possible thermoelectric applications. Thus, we first time make use of density functional perturbation theory (DFPT) calculations to determine the stable crystal structure via phonon dispersion. The electronic structure and thermoelectric properties are computed by density functional theory (DFT) and Boltzmann transport theory. To predict the overall physical (structural, electronic, mechanical, phonon) and thermoelectric properties, the paper is organized as follows.

## Computational methodology

The XTaZ (X = Pd, Pt and Z = Al, Ga, In) half-Heusler alloys are proposed to be crystallized in cubic F-43m structure. First, we perform geometry optimization and proper structural relaxation by fixing the volume relaxation of all these 6 materials. Monkhorst–Pack scheme for k-mesh size of 5 × 5 × 5 within an energy cutoff of 450 Ry is employed within QUANTUM ESPRESSO package^28^. The kinetic energy cutoff is fixed at 45 Ry for the plane-wave expansion of the electronic wave functions. We choose the Pedrew Burke Ernzerhof (PBE-GGA) correlation functional approximation^29^ with Rappe Rabbe Kaxiras Joannopoulos (ultrasoft) pseudopotential^30^. Keeping the convergence threshold of 10^−6^ Ry, SCF calculations are done within a cold smearing under the Methfessel-Paxton scheme set at 0.001 Ry. For phonon calculations, both DFT and DFPT simulations on a 5 × 5 × 5 q-mesh with a threshold of 10^−12^ are run for self-consistency. Boltztrap code is employed to calculate the transport properties^31^, but it furnishes the σ and κ_e_ in terms of τ. So, the coefficients are described by assuming the electron relaxation time of NiTiSn (τ = 1.5 × 10^−14^ s)^32,33^ and solving the electronic Boltzmann equation within this approximation. A large k-mesh of 50 × 50 × 50 was used for transport calculations. Slacks model is used to evaluate the lattice thermal conductivity of the present alloys^34–36^.

## Results and discussion

### Structure, phonon dispersion and stability

Chemically, it can be said that the group VB element Ta with high-lying atomic orbitals (5d orbitals) easily transfers the charge to the IIIA element (Al, Ga or In) in the present set of materials. However, d-filled atoms (Pd/Pt) preferably support the chemical bonding in an XTaZ molecule. The proposed XTaZ compounds are optimized in the conventional F-43m structure and the relaxed lattice parameters along with semiconducting energy gaps are listed in Table 1. In the nearest neighbor co-ordination, each X and Z atom forms XTa_4_ and ZTa_4_ tetrahedral structure, respectively. It is noteworthy to mention here that the interchanging of X and Y positions as XYZ and YXZ in actual crystal are crucial for electronic structure as they may exhibit semiconducting, metallic, or semi-metallic properties. We optimized both XTaZ and TaXZ structures and select the energetically most stable structure. The cohesive energies are of the order of 7.7 eV (PtTaAl), 5.9 eV (PtTaGa) and 4.9 eV (PtTaIn), 10.6 eV (PdTaAl), 5.5 eV (PdTaGa), 9.5 eV (PdTaIn). This energy predicts the phase stability and the bond strengths binding the respective atoms. However, the formation energies for Pt based alloys in order are − 2.04, − 2.85, − 1.08, eV and Pd based alloys are 0.81, 0.95, 0.27 eV, respectively. Since, the negative values of PtTaX alloys confirms the likeliness of their experimental synthesis but, the PdTaX group is unlikely metastable in nature due to small positive energies. Therefore, a novel Heusler material series is proposed in this report.

**Table 1:** Lattice constants (in Å), energy gaps (in eV) elastic constants and mechanical parameters (in GPa), Debye temperature (in K), compressional velocity (in m/s), sound velocity (in m/s) and average sound velocity (in m/s) as calculated for XTaZ alloys.

Parameter	PdTaAl	PdTaGa	PdTaIn	PtTaAl	PtTaGa	PtTaIn
Lattice constant (a_0_)	6.03	6.02	6.24	6.06	6.05	6.26
Band gap (eV)	0.31	0.46	0.33	0.56	0.95	0.92
**Lattice constants**						
C_11_	214.56	203.51	215.30	256.22	145.38	227.88
C_12_	124.30	136.09	114.97	136.00	237.98	118.57
C_44_	83.69	85.36	69.47	96.90	95.26	76.40
Bulk modulus (B)	154.3	158.56	148.42	176.07	176.24	155.00
Young’s modulus (Y)	171.70	156.87	160.90	208.44	188.43	175.25
Shear modulus (G)	65.32	58.81	60.98	80.01	71.31	66.81
Poisson’s ratio (υ)	0.31	0.33	0.31	0.30	0.32	0.31
Pugh’s ratio (B/G)	2.47	2.69	2.46	2.14	2.66	2.43
Cauchy pressure (C_12_–C_44_)	40.61	50.73	45.50	34.66	57.43	51.91
Compressional velocity (*v*_*l*_)	5045.35	4669.12	4543.18	4848.44	4508.83	4288.63
Sound velocity (*v*_*s*_)	2624.02	2326.02	2340.71	2579.14	2311.50	2243.65
Average sound velocity (*v*_*m*_)	2917.89	2575.43	2616.20	2870.34	2567.46	2504.85
Debye temperature (θ_D_)	329.43	291.61	286.96	322.87	289.10	272.630

**Figure 2:**
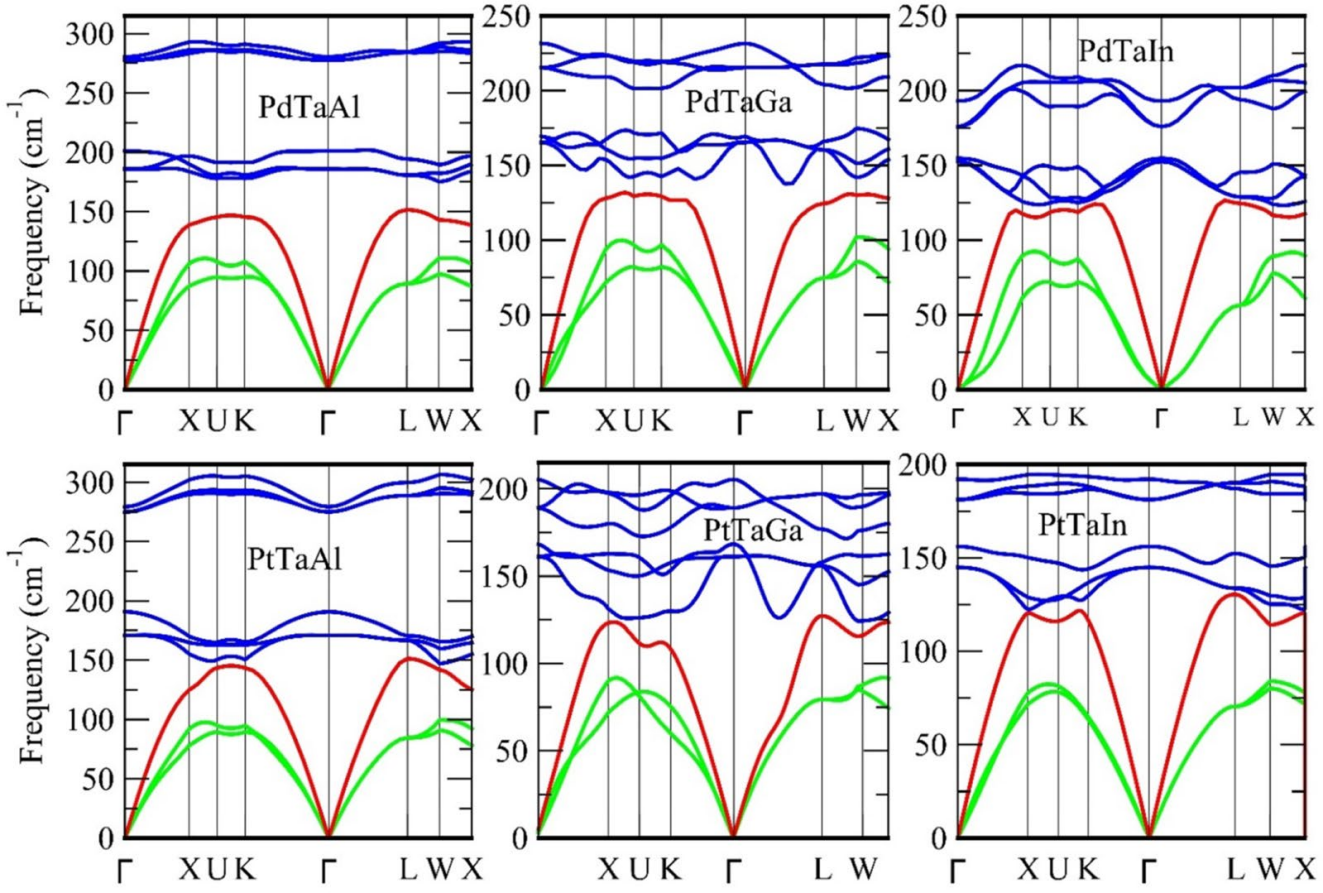
Phonon band structure of XTaZ alloys with no negative frequencies. Two green lines represent transverse acoustic (TA), red line indicates longitudinal acoustic (LA) and blue lines are six optical (O) modes.

The phonon dispersions are calculated to define the vibrational stability of the XTaZ alloys. The positive only phonon frequencies seen in Fig. 2 reflect the stable nature of these materials. Thus, XTaZ alloys are determined to be stable and to the best of our knowledge, these are not been reported in any database yet. Among the nine branches, low-lying three branches are acoustic (lower frequencies) and the six above in pairs of three each are optical modes (higher frequencies). Three degenerate regions are observed at Γ point, one acoustic and two optical regions. In all three regions, the transverse modes are doubly degenerate. The maximum acoustic phonon frequency of PdTaAl is 147.62 cm^−1^, PdTaGa is 135.24 cm^−1^ and PdTaIn is 148.75 cm^−1^ while as for PtTaAl, PtTaGa and PtTaIn alloys, the corresponding values are 151.8, 143.52 and 146.62 cm^−1^, respectively. The experimental thermoelectric material, NbFeSb alloy exhibits the maximum acoustic phonon at ~ 193.20 cm^−1^^21,37^. Acoustic modes in the present materials are located below ~ 150 cm^−1^ and the optic modes with small gap lie above that range. The optical modes largely being should contribute little towards the heat conduction. The acoustic phonon population is directly linked to the thermal conductivity at low temperature and its small value proves fruitful for less phonon scattering. Because, their maximum velocities majorly decide the lattice thermal conductivity^38^. Therefore, we can presume that the present materials will show less thermal conductivity in comparison to latter. The small gap or maximum overlap between acoustic (LA) and optical (TO) branches is observed which reduces the phonon scattering. In addition, it can be inferred that PtTaGa and PdTaGa may exhibit small conductivity in transport mechanism, which is verified in thermoelectric properties section. Also, the maximum optical mode frequency for all these materials is lower than ZrCoSb (285 cm^−1^)^39^, which implies the bonding is comparable to that of ZrCoSb^40,41^. This typical behavior plays a substantial role in deciding the transport properties of the XTaZ alloys.

### Elastic constants and mechanical properties

Keeping in view the structural stability via phonon dynamics and the mechanical standards of the present systems is discussed here. Voigt–Reuss–Hill (VRH) approximation is used^42–44^ for the elastic and mechanical properties analysis, and the subsequent equations are given below^45–47^.

1 $$ B_{V} = B_{G} = B = \frac{{(C_{11} + 2C_{12} )}}{3} $$

2 $$ G_{V} = \frac{{(C_{11} - C_{12} + 3C_{44} )}}{5};\quad G_{R} = \frac{{5(C_{11} - C_{12} )C_{44} }}{{4C_{44} + 3(C_{11} - C_{12} )}};\quad G = \frac{{G_{V} + G_{R} }}{2} $$

3 $$ {\text{Y}} = \frac{9BG}{{3B + G}};\quad \upsilon = \frac{3B - Y}{{6B}}{;}\quad A = \frac{{2C_{44} }}{{\left( {C_{11} - C_{12} } \right)}} $$

B, S, Y are bulk, Shear and Young’s modulus, respectively; A is the Zener anisotropy factor, υ is is considered as Poisson’s ratio. Later, the expedition of Debye temperature (*θ*_*D*_) is enlisted from the combinations of compressional velocity (*v*_*l*_), sound velocity (*v*_*s*_) and average sound velocity (ν_*m*_) as below;^48,49^

5 $$ v_{s} = \sqrt {\frac{G}{\rho }} \quad and\quad v_{l} = B = \sqrt {\frac{(3B + 4G)}{{3\rho }}} $$

6 $$ v_{m} = \left( \frac{1}{3} \right)^{\frac{1}{3}} \left( {\frac{2}{{v_{s}^{3} }} + \frac{1}{{v_{l}^{3} }}} \right)^{{ - \frac{1}{3}}} $$

7 $$ \theta_{D} = \frac{\hbar }{{k_{B} }}\left( {\frac{3n}{{4\pi }}\left( {\frac{{\rho N_{A} }}{M}} \right)} \right)^{\frac{1}{3}} v_{m} $$

where *ħ*, *n*, *k*_*B*_, *N*_*A*_, *M, ρ,* are the reduced Planck’s constant, number of atoms per formula unit, Boltzmann’s constant, Avogadro’s number, atomic mass, and density of unit the cell, respectively.

The mechanical stability is one among the concerned factors for choosing a material for desired applications. A material needs to be mechanically stable and practically feasible to be used in a thermoelectric module. Using the volume and energy conserving tetrahedral and rhombohedral distortions across the cubic structure, the various stability conditions taken into consideration are C_11_ + 2C_12_ > 0, C_11_ − C_12_ > 0, C_44_ > 0^50^. The various mechanical parameters listed in Table 1 are calculated from elastic constants using the relations mentioned in methodology section. The calculated Bulk, Young’s, and shear moduli of the present alloys increase first from Al to Ga and then decrease from Ga to In. Poisson's ratio (***ѵ***) hints the existence of central forces because ***ѵ*** > 0.25 < 0.50 for all the materials under study, while as 0.25 ≤ ***ѵ*** ≥ 0.50 classifies the non-central nature of bonding forces in a solid^51^. B/G (Pugh’s ratio) as well as accompanied by Cauchy pressure determine the ductile nature of all these Heuslers^52,53^. Using the data sets of various mechanical parameters, we predicted the Debye Temperature (**θ**_D_) of 329.43 K (PdTaAl), 291.61 K (PdTaGa), 286.96 K (PdTaIn), 323.58 K (PtTaAl), 278.66 K (PtTaGa), 278.08 K (PtTaIn), respectively.

### Electronic structure and density of states

**Figure 3:**
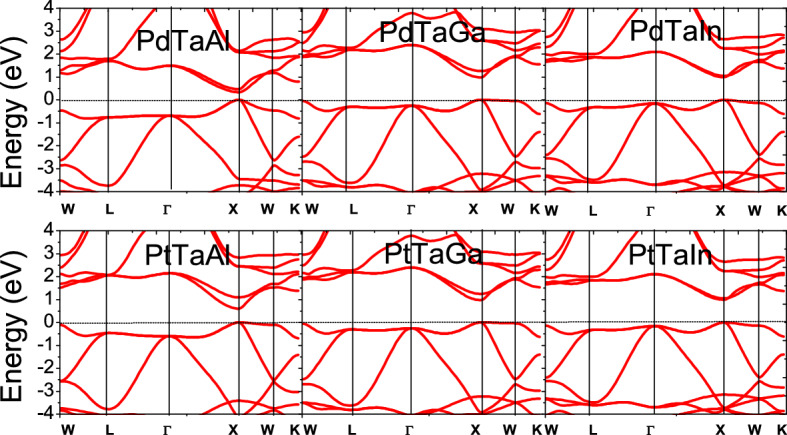
Electronic structure of XTaZ alloys with Fermi level set at zero.

The electronic structure of XTaZ alloys are investigated using the optimized lattice constants. All the three atoms in the present set of HH alloys exhibit T_d_ symmetry in their first coordination sphere and the octahedral symmetry in the next. Here we focus on the band structure along the high symmetry points as shown in Fig. 3. All these materials present a direct p-type band gap at X-point of the Brillion zone. PdTaAl, PdTaGa and PdTaIn show a gap of 0.31 eV, 0.46 eV and 0.33 eV, respectively; while as PtTaAl, PtTaGa and PtTaIn display larger gaps of 0.56 eV, 0.95 eV and 0.92 eV, respectively. In both these cases, this gap increases from Al to Ga but decreases again from Ga to In. This can be attributed to the addition of 5d-orbitals in In-based alloys, which increases the local effects due to extremely large size than that of the 3d-orbitals of Al-type materials. The top valence band exhibits flatness or band convergence, which is said to be favorable for large Seebeck power of these materials. Since, the flatness increases from Al to Ga therefore the Seebeck coefficient is supposed to improve accordingly due to amplified effective mass.

**Figure 4:**
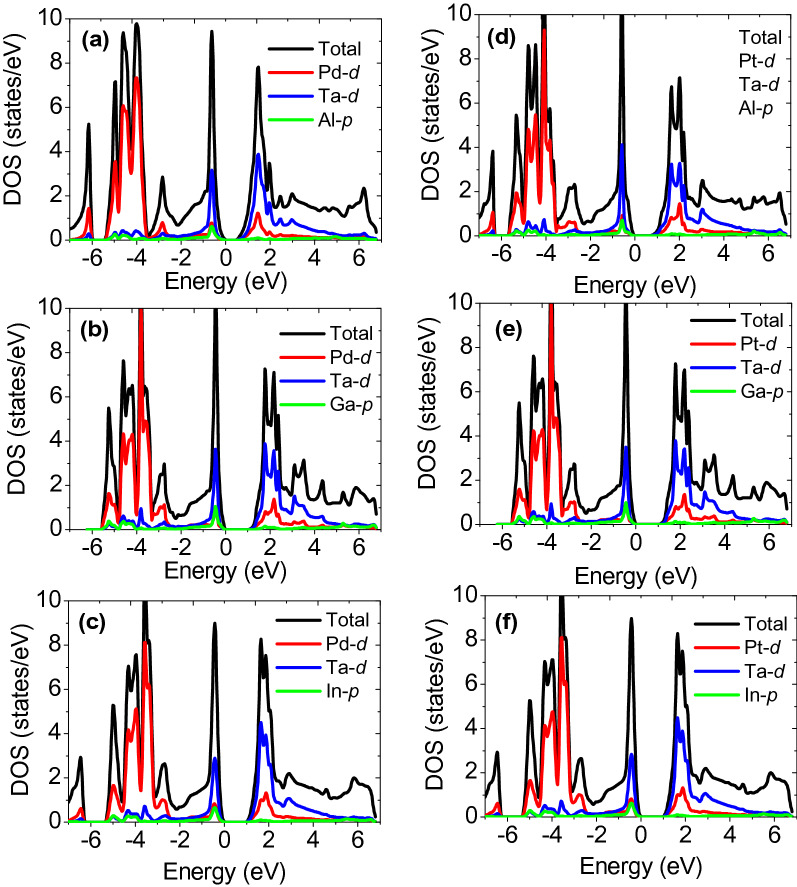
Projected (pDOS) density of states of XTaZ compounds.

The possible understanding of the semiconducting gap originates from the symmetry and position of constituent atoms, which thereby decide the occupation of bands around the Fermi level (EF). Total and partial density of states (TDOS, PDOS) presented in Fig. 4 are calculated to infer the strength of hybridization, occupation, and contribution from orbital states of individual atoms. XTaZ alloys display the semiconductor type band structure because of the generalized magic number-18 VEC rule^54,55^ which exhibits the 9 hybridized valence bands to fully compensate the spin effects in such systems. Here, we see the *d*-states of X and Ta atoms are mostly populating near the EF with an admixture of *p*-Z states. The orbital occupancies of individual atoms mix according to the octahedral *d-d* hybridization explained in ref.^56,57^. While interpreting the *p*-orbitals of Z atoms (Fig. 4a–f), it is observed that they occupy the low-lying bands over the energy range of 0–0.9 eV. However, the *d*-bands of Pd, Pt and Ta span from 1 to 4 eV in case of PdTaZ and 2–4 eV in PtTaZ alloys depicting their occupation and strength of hybridization. The two sets of bonding and antibonding orbitals formed from the split of *d*-bands in XTaZ alloys (each set from triple degenerate (*dxy, dxz and dyz*) orbitals and double degenerate *dx*^2^ − *y*^2^ and *dz*^2^ orbitals near the Fermi level) are responsible for the creation of band gap. Thus, a decisive new group of HH alloys is supposed to be displaying semiconducting properties.

### Thermoelectric coefficients

**Figure 5:**
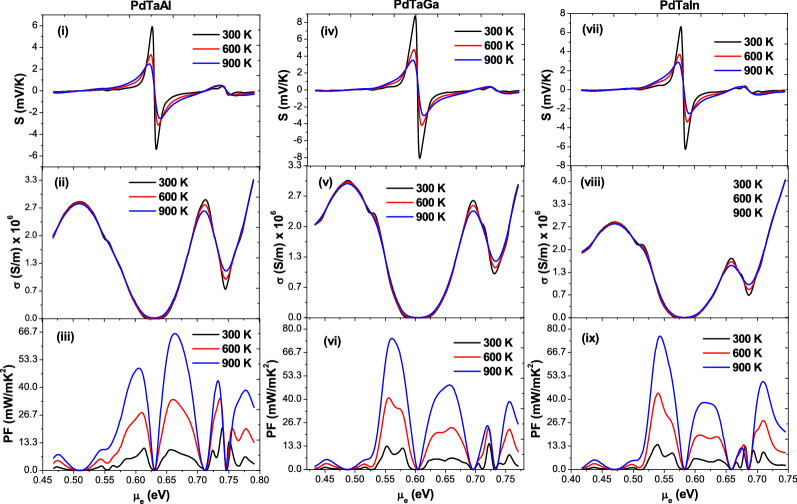
Transport coefficients of PdTaX(X = Al, Ga, In) alloys as a function of chemical potential: (**i**, **iv**,** vii**) Seebeck coefficient, (**iii**,** v**,** viii**) Electrical conductivity and (**iii**, **vi**,** ix**) Powerfactor.

**Figure 6:**
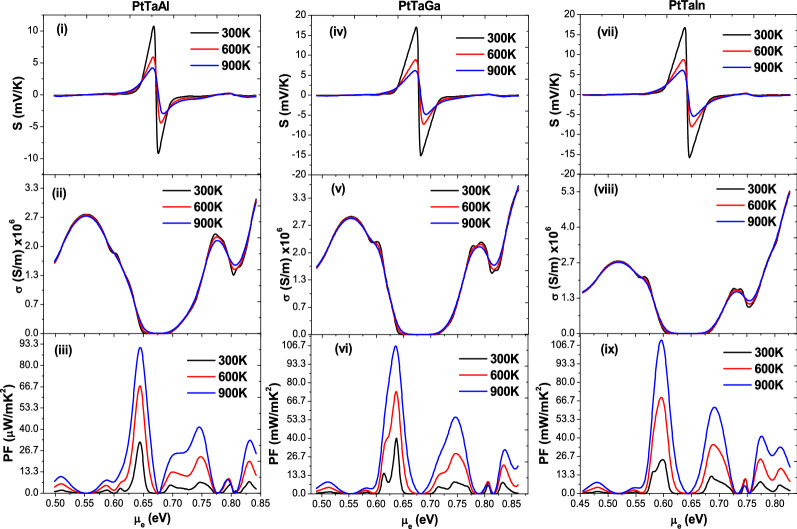
Transport coefficients of PtTaX(X = Al, Ga, In) alloys as a function of chemical potential: (**i**, **iv**,** vii**) Seebeck coefficient, (**iii**,** v**,** viii**) Electrical conductivity and (**iii**, **vi**, **ix**) Powerfactor.

**Figure 7:**
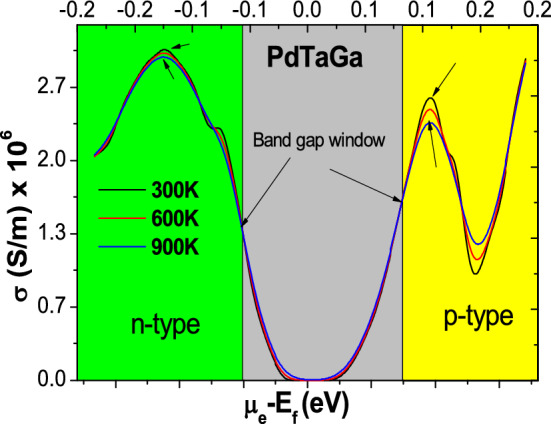
Electrical conductivity of PdTaGa (σ) as a function of chemical potential.

In Table 2, the Seebeck coefficient (S) and power factors (PF) are listed at 300 K and 900 K temperatures. At room temperature, the corresponding values of Seebeck coefficient are in the range of 1–3.5 mV/K in Pd-based alloys and 4–6 mV/K in Pt based alloys but interestingly higher temperature fluctuations reduce the magnitude by 5 times (see Figs. 5, 6). This type of behavior is true, mutatis mutandis for electrical conductivity (σ) against the same temperature gradient, even though the magnitude fluctuates within the smaller window of 2–3 × 10^−6^ S/m. It can be argued that the σ first increases as expected w.r.t **T** in the band gap region and then decreases away from it on both sides when hole or electron concentration magnifies (see the case of PdTaGa in Fig. 7). This is purely a semiconductor behavior when doped accordingly with n-type or p-type impurities. We also calculate the Powerfactor (PF) of the proposed alloys within the constant relaxation time approximation (see “Methods” for computational details). The large PF in these alloys is expected from the valley degeneracy due to location of CBM and VBM at low symmetry points and the d-orbital contributed high DOS near the Fermi level at the bottom of the conduction band and the top of the valence band seen in Figs. 3 and 4. Subsequently, the flat-and-dispersive band appearances (due to *d-*states) visualized near the Fermi level are in line with our expectations, which increases the PFs from ~ 10 mW/mK^2^ for PdTaAl to ~ 13.5 mW/mK^2^ for PdTaGa and up to 23–44 mW/mK^2^ for PtTaIn, PtTaAl and PtTaGa at 300 K (Figs. 5, 6, and Table 2). Similar type of increase in PF has been attributed to Fe-*eg* flat-and-dispersive bands in Fe_2_YZ alloys^58^. However, the present set of novel compounds unveil the massive upsurge in PFs from ~ 66 to 107 mW/mK^2^ at 900 K. In all Pd and Pt-based compounds exhibiting better TE properties at higher temperatures, the PF rests on substantial magnitude (i.e. 95% peak) in a comparable and reasonably wide range of carrier concentrations (n ≈ 1 × 10^20^–2 × 10^21^ cm^−3^) at which |S| ~ 1.5–6.0 mV/K. In addition, comparable to the best available TE Heusler Fe_2_Val (4–6 mW/mK^2^)^59^, the present alloys then display large S at higher temperatures. Thus, in XTaZ compounds, the improved TE properties at ideal doping concentrations are expected instead by electrons from the transition metal d-eg lowest-conduction states and are possibly anticipated in finding applications in high temperature TE materials.

**Table 2:** Approximate values of Seebeck coefficient (S) and Powerfactor (PF) of XTaZ alloys calculated at 300 K and 900 K temperature range.

Parameter	Temp	PdTaAl	PdTaGa	PdTaIn	PtTaAl	PtTaGa	PtTaIn
S (mV/K)	300 K	1.5	3.5	2.8	4.0	6.0	5.0
PF (mW/K^2^)		11.0	13.5	12.6	33.5	40.0	23.4
S (mV/K)	900 K	6.0	8.5	6.5	11.0	17.0	16.5
PF (mW/K^2^)		66.5	76.5	74.4	90.5	106.7	106.5

### Lattice thermal conductivity

**Figure 8:**
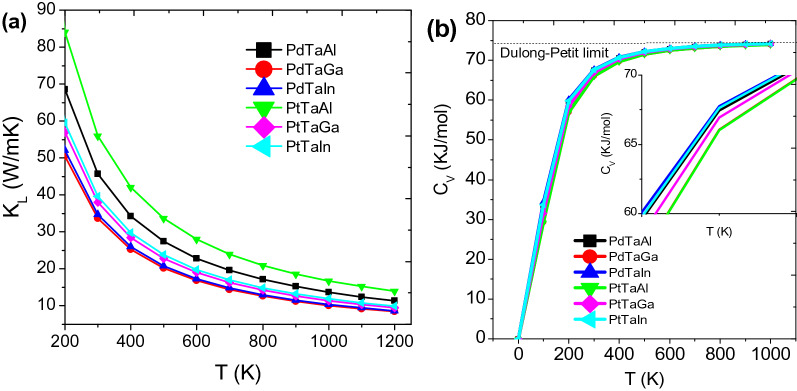
(**a**) Lattice thermal conductivity (*κ*_*L*_) and (**b**) Specific heat capacities of XTaZ compounds as a function of temperature.

Lattice thermal conductivity is a prerequisite property decided by the phonon spectrum and the phonon-scattering types and rates, their temperature or frequency dependencies, Gruneisen parameters, etc.^33,60,61^. Focusing on the intrinsic thermal conductivity only particular to a specific temperature range, we suppose anharmonic Umklapp (resistive phonon–phonon scattering where the total crystal momentum is not conserved) processes are very important to govern the interactions among the phonons. There occurs another non-resistive phonon scattering process where the conservation of total crystal momentum is observed known as the normal or N-process. This consequences the indirect contribution to the thermal resistance by N-process, although they effectively transfer energy between different phonon modes. Since, Slack-model mainly focuses on high temperature; N-process occurring frequently at lower temperatures are ignored. Because at low temperatures, N-process is compensated by mass fluctuation and strain field terms in Heusler alloys^62^. Thus, the typical dominance of U-process at higher temperatures when scattering enabled by energy occurs beyond the first Brillouin zone. Therefore, we can say the bulk acoustic phonons are the dominant heat carriers in this context. This can be observed from the comparative analysis of XTaZ alloys in Fig. 8a, where PtTaAl (147.62 cm^−1^) and PdTaAl (151.80 cm^−1^) with higher values of acoustic phonon frequencies are having large thermal conductivity values in comparison to the PdTaGa (135.24 cm^−1^) and PtTaGa (143.52 cm^−1^), respectively. The room temperature values of XTaZ alloys are 45.6 W/K (PdTaAl), 33.6 W/K (PdTaGa), 34.6 W/K (PdTaIn), 56.1 W/K (PtTaAl), 38.0 W/K (PtTaGa), 39.6 W/K (PtTaIn), respectively. However, these values are quite large in comparison to available thermoelectric materials. For example, κ_L_ for NiTiSn is 9.3 W/mK at 300 K^33^ and for Fe_2_Val is = 28 W/mK at 300 K^59^. Recently, Feng et al.reported PtLaSb (0.84 W/mK), RhLaTe (1.21 W/mK) as the minimum and IrSnTa (78.09 W/mK) as the maximum thermal conductivity materials among 74 half-Heusler alloys^60^. Thus, to further the research in having possible TE applications, lattice thermal conductivity needs to be reduced in such alloys and could possibly be achieved via 2D approaches or alloying/doping^63^. It is well established from experiments and theoretical observations that including vacancy-antisite scattering, grain-boundary scattering, etc.^61^. More importantly, the avoided crossings of the optical phonon and longitudinal acoustic branches are a characteristic of rattling in these alloys. For low thermal conductivity materials, the low frequency phonons are scattered at faster rates. Thus, anharmonic scattering rates, (anharmonicity and the scattering phase space) are clearly significant for thermal conductivity.

### Specific heat capacity

Specific heat measurements for XTaZ alloys w.r.t high temperature is entertained by using the quasi-harmonic Debye model^49,51,64^. First, the different total energy versus volume data sets are fitted in static approximation with equation of state (EOS) to predict the crystal parameters at ambient conditions (P = 0 and T = 0). Later, the standard thermodynamic relations are utilized to obtain the macroscopic characteristics as a function of temperature. In Fig. 8b, the specific heat capacities of the alloys under study are plotted as a function of temperature. At low temperatures, C_V_ is independent of T and therefore increases until it reaches the Dulong-Petit limit^65^. The unavailability of experimental values of C_V_ bars us to compare the data and hence, the approximate Dulong-Petit limit is referenced to be around 75 kJ/mol.

## Conclusion

To conclude, the structural, mechanical, spintronic properties and phonon dynamics of new HH materials has been successfully investigated in this report. *F-43m* structural symmetry is adopted by these alloys and is dynamically and energetically favored configurations. Electronic structure reveals the semi-conductive nature with energy gaps in the visible range. Elastic calculations describe the mechanical stability of the presumed structures as well as ductile nature. Detailed analysis of material properties in this report pave the way to design novel materials with desired properties experimentally. In the end, the mechanical stability as well as ductile nature is determined, where in the Debye temperature of all these compounds is observed to be within the room temperature range. Our predictions will stimulate extra efforts in experimental and theoretical domains to investigate the possible thermoelectric applications of HH alloys.
